# Quantitative Assessment of Response to Long‐Term Treatment with Intravenous Immunoglobulin in Patients with Stiff Person Syndrome

**DOI:** 10.1002/mdc3.13261

**Published:** 2021-06-18

**Authors:** Smriti Bose, Joseph P. Thompson, Girija Sadalage, Abid Karim, Saiju Jacob

**Affiliations:** ^1^ Department of Neurology University Hospitals Birmingham Birmingham United Kingdom; ^2^ Clinical Immunology Service University of Birmingham Birmingham United Kingdom; ^3^ Institute of Immunology and Immunotherapy University of Birmingham Birmingham United Kingdom

**Keywords:** stiff person syndrome, PERM, GAD, glycine, BRIT

## Abstract

**Background:**

Stiff person syndrome (SPS) is an autoimmune condition involving antibodies against several components of the inhibitory synapse in the spinal cord, with glutamic acid decarboxylase antibodies being the predominant immune marker. SPS affects approximately 1 patient per million population per year. The effect of intravenous immunoglobulin (IVIG) has been established, but studies on the long‐term efficacy of regular IVIG are limited.

**Objectives:**

To review clinical details and long‐term treatment response using a patient‐reported questionnaire in SPS and related syndromes.

**Methods:**

Patients were identified from a tertiary neuroimmunology clinic based on classical clinical symptoms, autoimmune profiles, and neurophysiological changes (Dalakas criteria). They were followed up after treatment to assess the response to IVIG.

**Results:**

A total of 23 patients fulfilled the selection criteria. Patients' demographic profiles and clinical presentations were akin to that reported in literature. There was significant improvement in the functional ability (assessed by the modified Rankin scale [mRS]) and quality of life (QoL) following treatment with IVIG within 4 to 10 weeks (pre‐mRS vs. post‐mRS, *P* < 0.0001; pre‐QoL vs. post‐QoL, *P* = 0.0003) and sustained after 5 years of treatment (pre‐mRS vs. present mRS, *P* = 0.0003; pre‐QoL vs. present QoL, *P* = 0.0002).

**Conclusions:**

This article describes one of the largest single‐center experiences of 23 patients with SPS and related syndromes and is the first to establish the long‐term efficacy of regular IVIG using a patient‐reported scoring system (Birmingham Response to Immunomodulatory Therapy [BRIT]). Consistent improvement in QoL and functional scores were seen over nearly 5 years after regular use of IVIG. It is recommended to use BRIT scores to assess the initial response as well as to monitor continued improvement to immunomodulation in SPS.

Stiff man syndrome was first described in 1956 by Moersch and Woltman before being redefined as stiff person syndrome (SPS) in 1991 after acknowledging the prevalence of the condition in women, in whom it predominates.^1^, ^2^ It is a rare autoimmune condition with an incidence of 1 patient per million per year.^3^ Various antibodies against several components (Fig. 1A) of the inhibitory synapse have been implicated (although only glutamic acid decarboxylase [GAD] and glycine antibodies are routinely tested and practically useful), resulting in the core clinical presentation of fluctuating muscle stiffness and spasms with significant impairment in the quality of life (QoL).^4^ Diagnosis therefore requires a high index of suspicion.

**FIG. 1:**
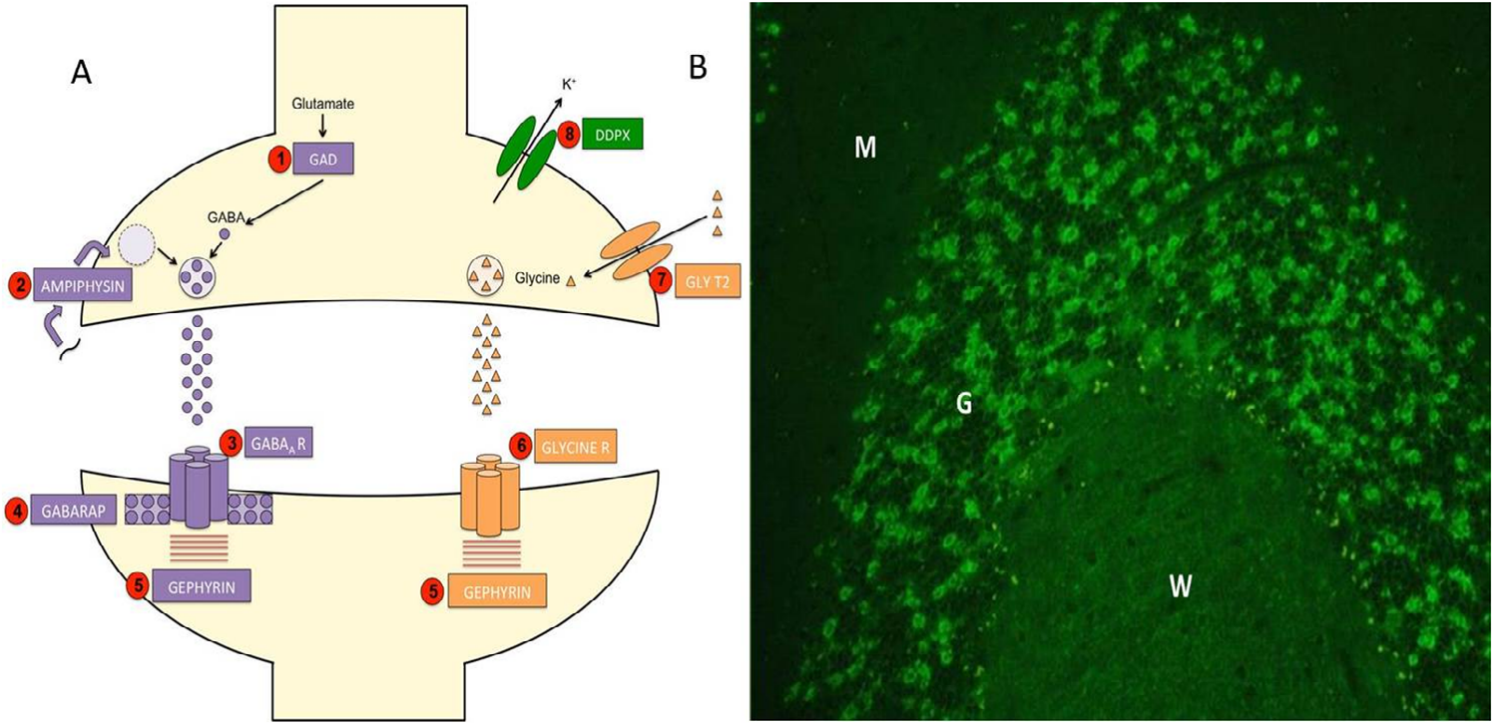
(**A**) Stiff person syndrome (SPS) antibody targets along with other proteins in the inhibitory synapses. (1) Glutamic acid decarboxylase (GAD) is responsible for the formation of gamma‐aminobutyric acid (GABA). (2) Amphiphysin recycles GABA vesicle membranes. (3) The GABAA receptor controls the inhibitory signaling. (4) GABAA receptor associated protein (GABARAP) provide structural support to the GABAA receptor. (5) Gephyrin is involved with protein clustering at both the GABAA receptor and (6) the glycine receptor. (7) Glycine transporter 2 (GLY T2) recycles glycine from the synapse. (8) Dipeptidyl‐peptidase‐like protein‐6 (DDPX) regulates Kv4.2 potassium channels. Currently only GAD and glycine antibodies are practically useful for clinical management, with amphiphysin antibodies seen predominantly in the paraneoplastic variants. Although the other antigens have been reported as targets in isolated case reports, these have not been replicated consistently. (**B**) Immunofluorescence staining produced by GAD antibodies. Patchy staining of neuropil in the granular layer (nerve terminals) can be visualized. For orientation purposes, the Purkinje cell can be found on the border of molecular layer (M) and granular layer (G), which surrounds the white matter of the cerebellum (W).

We describe the common presentations of SPS in one of the largest case series from a single center and bring forward an objective assessment of long‐term response to treatment with repeated intravenous immunoglobulin (IVIG) infusions using a patient reported questionnaire: the Birmingham Response to Immunomodulatory Therapy (BRIT) scale. We also compared the functional ability of patients who did not receive long‐term IVIG to the group who received it.

## Methods

The study included patients from a tertiary neuroscience center based on clinical, neurophysiological and immunological criteria. Patients were excluded if the presentation was not considered typical of an autoimmune phenotype despite the antibodies or there was an alternative explanation for the symptoms. Data were retrospectively collected by analyzing clinic letters, laboratory results, and electromyography (EMG) reports. The BRIT scale was filled in 3 phases. The time duration between the first (pre‐IVIG) and second phase (post–IVIG) was 4 to 10 weeks. The third phase (latest clinical status) was done approximately 3 to 5 years later.

The BRIT scale includes a functional ability score and a QoL score. Functional ability was assessed with the modified Rankin scale (mRS) in which patients must score their best and worst functional abilities at the time of filling the questionnaire and during the past 3 months (or since the last cycle of IVIG). The scores are on a scale of 0 = “no symptoms” to 5 = “severe disability” (higher score representing more disability). QoL was assessed using a questionnaire based on the Flanagan's scoring system in which patients answer 16 questions and rate each answer on a 7‐point scale in which 1 = “terrible” and 7 = “delighted.”^5^ The total sum of these 16 questions gives the final QoL score (maximum score, 112; higher scores shows better QoL). Patients filled out the questionnaires (with the physical assistance of staff or family members if needed) on the day that they received the IVIG. The pre‐BRIT, post‐BRIT, and current BRIT scores were then analyzed. The patients who did not have an objective improvement (1 point decrease in mRS and 8 point increase in QoL) following an average of 3 courses of IVIG were advised to discontinue treatment. The most recent mRS scores of these patients were compared with patients who received regular IVIG.

Data were analyzed using paired and unpaired *t* tests, and graphs were generated using GraphPad (GraphPad Software, San Diego, CA).

## Results

A total of 23 patients were identified and classified as SPS (n = 15), SPS plus (n = 2), progressive encephalomyelitis with rigidity and myoclonus (PERM; n = 2), focal SPS (n = 2), or other GAD syndromes (n = 2). Patient demographics and laboratory features are summarized in Table 1.

**TABLE 1:** Summary of demographics, laboratory features, and treatment

Patient No.	Age, y	Sex	Diagnosis	Antibody Target	EMG Features	Time Since Onset of Symptoms to Diagnosis, y	Time Since Symptom Onset to Treatment with IVIG	GABAergic Drugs Benzodiazepines/Baclofen	Current Immunotherapy
1	41	Female	SPS	GAD	No	5	7	Yes/Yes	IVIG
2	33	Male	SPS	GAD	Unknown	Unknown	Unknown	Yes/Yes	IVIG
3	40	Female	Focal SPS	GAD	No	6	6	Yes/No	IVIG
4	44	Female	SPS	GAD	No	8	8	Yes/Yes	Discontinued after 2 courses
5	44	Female	SPS	GAD	Yes	<1	0	Yes/Yes	IVIG
6	68	Female	SPS	GAD	Yes	1	2	Yes/Yes	IVIG
7	38	Male	SPS	GAD	Yes	<1	0	Yes/No	IVIG
8	45	Female	SPS	GAD	Unknown	<1	2	Yes/Yes	IVIG
9	28	Female	SPS		Yes	6	11	Yes/Yes	Discontinued after 3 courses
10	36	Female	SPS Plus	GAD	No	6	8	Yes/Yes	Discontinued after multiple courses
11	39	Female	SPS	GAD	No	2	3	No/No	IVIG
12	36	Female	SPS	GAD	No	6	6	Yes/Yes	IVIG
13	33	Male	SPS	GAD	Unknown	<1	21	Yes/Yes	Discontinued × 1 course
14	52	Male	SPS Plus	GAD	No	1	1	No/Yes	IVIG
15	59	Female	SPS	GAD	Unknown	<1	2	Yes/No	SCig
16	51	Male	SPS	GAD	Yes	<1	1	Yes/Yes	Discontinued after 2 courses
17	47	Female	SPS	GAD	No	1	1	Yes/Yes	IVIG
18	59	Female	Focal SPS	GAD	Yes	6	6	No/No	IVIG
19	46	Female	PERM? Paraneoplastic	Glycine	No	<1	2	No/Yes	Discontinued after single course
20	42	Male	PERM	Glycine	Unknown	25	27	No/Yes	IVIG
21	51	Female	SPS	Glycine	No	<1	1	No/Yes	IVIG
22	59	Male	Cerebellar ataxia	GAD	No	<1	1	No/No	IVIG
23	65	Female	Cerebellar ataxia	GAD	Unknown	Unknown	Unknown	Yes/No	IVIG

Abbreviations: EMG, electromyography; IVIG, Intravenous Immunoglobulin; SPS, stiff person syndrome; GAD, glutamic acid decarboxylase; PERM, progressive encephalomyelitis with rigidity and myoclonus; SCIg, subcutaneous immunoglobulin.

Mean age (± SD) at presentation was 46 ± 10.66 years (range, 28–68 years). The male:female ratio was 1:2.3. Of the patients, 7/23 (30.4%) affirmed they were of mixed or Afro‐Caribbean/Black descent, and 14/23(60.9%) were of White descent. Earliest presentation was spasms, most often in the lower limbs. Frequency of presentation of various clinical phenotypes is given in Table 2.

**TABLE 2:** Clinical phenotypes in patients with stiff person syndrome and their frequencies

Clinical features	Absolute Numbers	Percentage
History of falls	20/23	87
Balance difficulty	17/23	73.9
Spasms	17/23	73.9
Pain	16/23	69.6
Exaggerated startle response	15/23	65.2
Increased tone/brisk reflexes	15/23	65.2
Psychiatric illness	13/23	56.5
Other autoimmune condition	8/23	34.8
Speech difficulty	6/23	26.1
Abnormal eye movements	5/23	21.7
Epilepsy	4/23	17.4
Ataxia	4/23	17.4
Dyspnoea	1/23	4.3
Dysphagia	1/23	4.3
Neoplasia	1/23	4.3
Appetite changes	0/23	0

Unsteadiness, stiffness, pain, and exaggerated startle responses were the most common symptoms. Of the patients, 19/23 (82.6%) had positive GAD antibodies, with 3/23 (13.0%) having glycine receptor antibodies; 1 was seronegative (4.3%). Typical SPS EMG changes of continuous motor unit activity was seen in 5/15 patients (33.3%).

The average time to diagnosis and start of IVIG treatment since symptom onset was 3.5 years and 5.3 years, respectively. Of the patients, 17/23 (73.9%) are currently on immunomodulation, with 1 on subcutaneous immunoglobulin therapy. The time between infusions was determined by the loss of clinical efficacy (assessed both subjectively and objectively) and varied between 4 weeks to 6 months.

### BRIT Scores

**FIG. 2:**
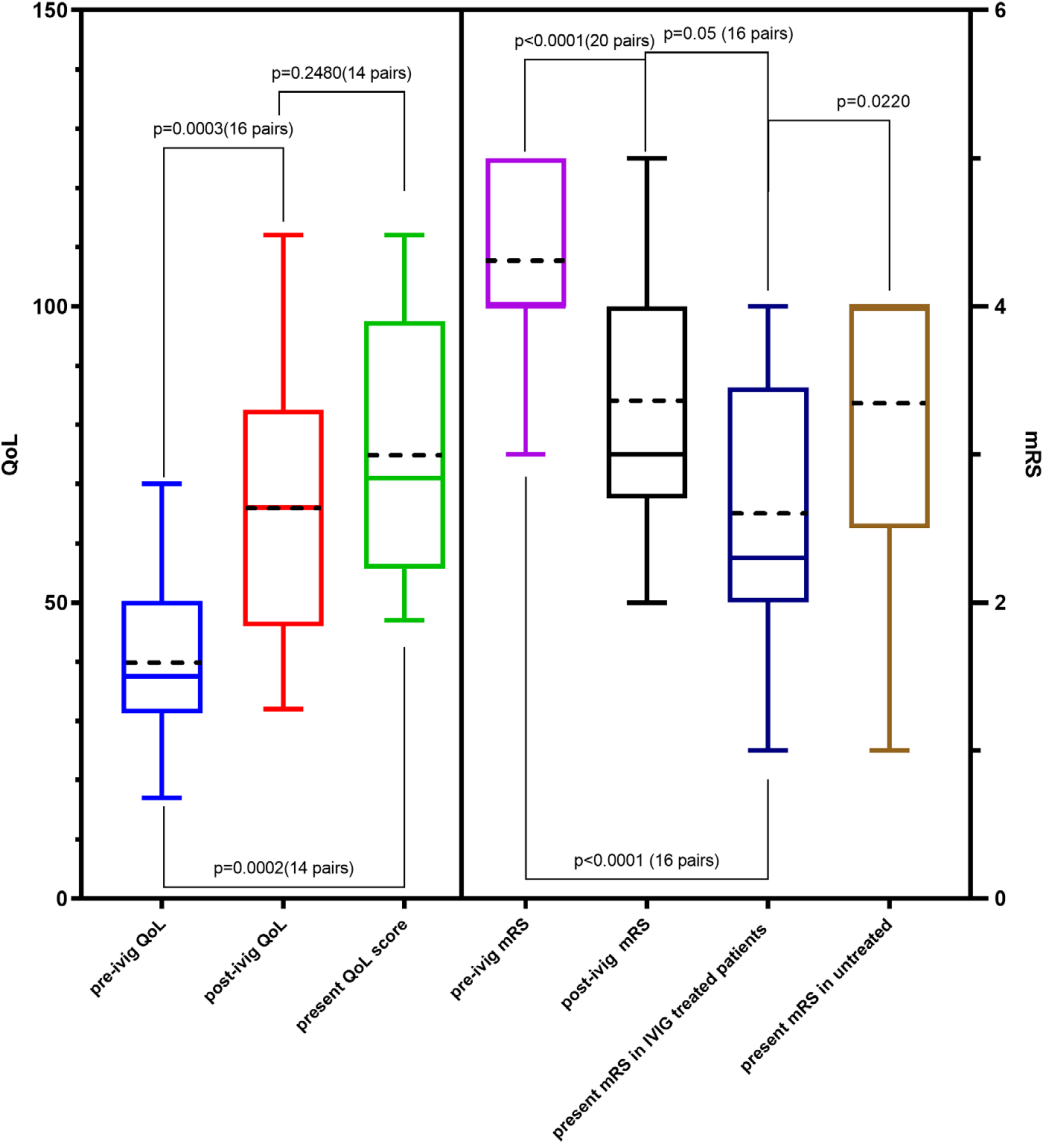
Response to immunomodulatory therapy after intravenous immunoglobulin (IVIG). Quality of life (QoL; left) and functional ability score (modified Rankin scale [mRS]; right) before and after the administration of IVIG treatment compared with the latest scores. Flanagan's QoL scoring system ranges from a total minimum of 16 points (which would represent “terrible” in all 16 categories) to a possible maximum of 112 points (which would represent “delighted” in all 16 categories). Higher QoL scores suggest subjective improvement. On the mRS scale, 0 = no symptoms and 5 = severe disability. Lower mRS scores suggest better functional outcome. At the latest time point, patients treated with IVIG had significantly better functional ability scores compared with those who did not have regular IVIG (*P* = 0.022). Mean shown as dotted line and median as solid horizontal line.

Significant improvements in the functional ability score (*P* < 0.0001) and QoL score (*P* = 0.0003) were recorded after IVIG infusion. These improvements were seen soon after the initial course and were sustained during a period of 5 years of regular IVIG infusions (Fig. 2). However, there was no significant difference recorded between immediate post‐IVIG and present BRIT scores, suggesting sustained response. The mRS scores of patients on regular IVIG were significantly better (*P* = 0.022) compared with those not on immunomodulation (Fig. 2).

## Discussion

This is one of the largest cohorts of patients with SPS spectrum disorder from a single center with regular follow‐up for up to 5 years. Most patients were positive for GAD antibodies in keeping with published literature, with the 2 patients with PERM being positive for glycine receptor antibodies.^6^

The demographics and clinical presentation of SPS in our cohort was similar to what has been described in literature.^4^ More than 90% of our cohort presented with frequent falls, imbalance of gait, and spasms. Not all patients fulfilled the Dalakas criteria, in particular the EMG criteria.^3^ It is therefore imperative to have a high index of suspicion in patients presenting with similar complaints. The presence of an underlying psychiatric illness was high (seen in 57% of our cohort) and may lead to misdiagnosis as a functional disorder, in which case clinical findings of increased tone and reflexes maybe useful indicators of pathology. It is unclear whether some of the psychiatric symptoms (depression, anxiety, etc.) may have been secondary to the long delay from symptom onset and diagnosis or directly related to the neurotransmitter abnormalities. It is important to use a combination of clinical symptoms, examination findings, antibody status, and neurophysiology to avoid inaccurate diagnoses. A diazepam challenge may also assist in diagnosis.^3^, ^7^ We initiated IVIG in patients unresponsive to GABAergic drugs or with worsening symptoms despite symptomatic therapy. It was continued only if objective clinical improvement was noted. The clinical profile of the untreated patients was progressive, whereas regular IVIG seemed to not just stabilize but improve BRIT scores in the long term. A longitudinal study of 57 patients done during an 8‐year follow‐up confirmed the progressive nature of the disease.^8^ About 69% of our patients on regular IVIG had a 5‐year follow‐up after phase 2, whereas the others were followed up for at least 3 years. SPS is a chronic progressive condition, and our study suggests that patients responsive to IVIG are likely to have sustained clinical improvement despite the progressive nature of the illness. Patients unresponsive to IVIG, however, have variable and more complex outcomes with a higher chance of worsening disability.

Understanding the pathophysiology of the disease and information from long‐term follow‐up data may help develop treatment protocols. GAD is an enzyme essentially involved in the rate‐limiting step in the formation of gamma‐aminobutyric acid (GABA). Antibodies against it are likely to decrease GABA. Antibodies to GABA receptors have also been reported in up to 65% of patients with SPS.^3^ This could explain the benefit of GABAergic drugs (eg, benzodiazepine, baclofen) in symptom control. Patients with concurrent psychiatric illness also stand to benefit from benzodiazepines because of their anxiolytic properties. These drugs are, however, associated with tolerance and withdrawal effects.

There are only 2 large‐scale randomized control trials so far looking at effective choice of treatment, namely, rituximab and IVIG.^9^, ^10^ In both trials, patients were continued on GABAergic drugs—baclofen, benzodiazepines, and so on. Although Dalakas reported transient benefit (6 weeks to 1 year) following a single course of IVIG,^9^ the benefit of rituximab as an effective therapy (at 6 months) in a similar trial could not be established.^10^ Intermittent shortage of IVIG supply in the past has led to restrictions intended to safeguard supplies. This led to a need for regular approvals for IVIG. Mode of administration that requires patients with restricted mobility to make frequent visits to the hospital along with well‐established adverse effects are also a hindrance to its regular long‐term use. GABAergic drugs tend to act as bridging therapy until circumstances (tolerance, adverse effects, and severe disease) require definite management with IVIG. Our study also proves that significant improvement is likely to occur despite delayed IVIG therapy. Jones and colleagues reported a similar fluctuating transient response with the progression of disease in a 50‐year‐old woman treated with IVIG for 2 years.^11^ One study reported sustained clinical improvement in 98/99 patients on at least 1 GABAergic drug with additional improvements in only 41% of those who received immunotherapy, including IVIG.^12^ Muñoz‐Lopetegi and colleagues^13^ compared clinical relevance and clinical and serological responses to treatment in patients with high and low GAD antibody concentrations. In the high antibody concentration group with associated neurological syndromes, 70% improved with immunotherapy. The improvement tended to stagnate after initial improvement. None of the patients had complete recovery, and a recurrence of symptoms were noted once immunotherapy was stopped.^13^ The lack of significant improvement in the post‐IVIG and present BRIT scores in our study could be attributed to either progression of the disease, a “ceiling effect,” or a lack of longer term follow‐up data in roughly 31% of our patients. We are continuing to monitor the response at periodic intervals, which is also now a requirement for IVIG approval.

Patients on IVIG had comparatively better scores at the latest assessments than those who were not. A possible reason could be the natural progression of immune therapy‐resistant patients as opposed to those responsive in whom disease progression had slowed down.

This study has therefore established the benefits of regular IVIG in patients with severe disease not responding to the conventional symptomatic therapy. This has not been investigated earlier. The BRIT scale provides a uniform assessment tool with minimum observer bias (as the patient is the only assessor) in a condition with variable disease course and response to treatment. One criticism would be that this was a subjective placebo response. However, to have consistent improvement sustained over 3 to 5 years in a large numbers of patients would be against this. Also, patients did not have access to their previous scores, and hence it is unlikely that this would have influenced the subsequent scores. The distribution of the stiffness index and the heightened sensitivities scale used by Dalakas^9^ requires the same neurologist to do it. This may not always be feasible in a long‐term study. Because the initial use of the BRIT score in our center several years ago,^14^, ^15^, ^16^ the UK immunoglobulin approval guidelines recommend this as one of the criteria for monitoring IVIG efficacy assessment in patients with SPS.^17^ Other scoring systems have several limitations, for example, startle response (not all patients may have startle all the time), timed walking test (unable to do in nonambulant patients), and so on.

A study by Gerschlager and colleagues in 2002 reported improvement in the QoL following IVIG in 6 patients with classical SPS. The Medical Outcomes Study Short Form Health Survey and the visual analog scale were used for this purpose.^18^ The BRIT assesses both functional mobility and its effect on QoL. It can also be done via a telephone interview, considering the limitations of face‐to‐face assessments during the COVID‐19 pandemic.

From our experience, we propose that at least a 1‐point improvement in the functional ability score and an 8‐point improvement in the QoL score are needed before considering that a patient is IVIG responsive. As in inflammatory neuropathies, we recommend a trial of at least 3 infusions (unless limited by adverse effects) before considering a patient to be a nonresponder. Our previous work had compared these scores in patients with typically IVIG responsive conditions such as chronic inflammatory demyelinating polyneuropathy, and the scores correlated well with other clinical assessment parameters.^15^ Further work may need to be done in a larger cohort of patients across different centers to ensure reproducibility and validation.

The strengths of this study are the number of patients we could analyze during a period of roughly 5 years and the use of an easy‐to‐administer tool for quantitative assessment. This is the first study to quantitatively measure long‐term efficacy of regular IVIG in patients with SPS. The limitations, however, are the lack of a comparable placebo arm, the variable time period between assessments, and recall bias in some patients who were assessed retrospectively.

To conclude, there was consistent improvement over time with regular IVIG use in nearly three quarters of the patients with SPS, although it is still unclear whether IVIG should be used as first‐line therapy before attempting symptomatic therapy such as benzodiazepines and baclofen. However, the autoimmune nature of SPS is established beyond doubt, and immunomodulation is likely to be needed sooner rather than later. We recommend the use of BRIT scores for measuring the response to IVIG in SPS. We aim to conduct future studies to formulate management guidelines with the help of BRIT scores.

## Author Roles

(1) Research Project: A. Conception, B. Organization, C. Execution; (2) Statistical Analysis: A. Design, B. Execution, C. Review and Critique; (3) Manuscript Preparation: A. Writing of the First Draft, B. Review and Critique.

S.B.: 1B, 1C, 2A, 2B, 2C, 3A, 3B

J.P.T.: 1B, 1C, 2A, 2B, 2C, 3A, 3B

G.S.: 1B, 1C, 2A, 2B, 2C, 3B

A.K.: 1B, 1C, 2C, 3B

S.J.: 1A, 1B, 1C, 2A, 2B, 2C, 3A, 3B

## Disclosures

### Ethical Compliance Statement

This was a retrospective case note review and a formal ethical or research approval was deemed un‐necessary. All patients consented to take part in the questionnaire assessment and no patient identifiable data has been submitted with this manuscript. We confirm that we have read the Journal's position on issues involved in ethical publication and affirm that this work is consistent with those guidelines.

### Funding Sources and Conflicts of Interest

None of the authors have any funding sources or conflict of interest relevant to the study.

### Financial Disclosures for the Previous 12 Months

Saiju Jacob has served as an international advisory board member for Alexion, ArgenX, Regeneron, Immunovant and UCB pharmaceuticals, is currently an expert panel member of Myasthenia Gravis consortium for Argenx pharmaceuticals and has received speaker fees from Terumo BCT, UCB, ArgenX and contentednet.com. Girija Sadalage has served on the expert Myasthenia Gravis panel for PRMA consulting. Smriti Bose, Joseph P Thompson and Abid Karim have no financial disclosures/conflicts of interest to declare.
